# Premature mortality and years of life lost attributable to ambient air pollution in Israel, compared to Europe: analysis and implications

**DOI:** 10.1186/s13584-026-00753-4

**Published:** 2026-03-09

**Authors:** Ilan Levy, Itamar Grotto, Hagai Levine, Isabella Karakis

**Affiliations:** 1 Air pollution and asbestos prevention division, Ministry of Environmental Protection, Jerusalem, Israel; 2 School of Public Health, Ben Gurion University of the Negev, Beer Sheva, Israel; 3 Braun School of Public Health and Community Medicine, Hadassah Medical Center, Faculty of Medicine, Hebrew University, Jerusalem, Israel; 4 Environmental Epidemiology, Public Health Division, Israel Ministry of Health, Jerusalem, Israel

**Keywords:** Environmental health risk, Particulate matter, PM2.5, Premature death, Mortality, Health effects, Counterfactual concentrations

## Abstract

Exposure to air pollution is considered the most significant environmental risk factor to human health. Updates in epidemiological studies in recent years provided the scientific evidence for the World Health Organization (WHO)’s air quality guidelines in 2021, allowing an update to the quantitative calculation of premature death (PD) from air pollution. This study examines the impact of air pollution on public health in Israel, focusing on PD and years of life lost (YLL) associated with exposure to PM_2.5_, nitrogen dioxide (NO_2_), and ozone (O_3_) and compares them to findings from 41 European countries. Applying the latest WHO air quality guidelines and incorporating census tract level demographic with age and gender separation, the study reveals the significant burden of air pollution in Israel. Between 2015 and 2023, a total of 4,461–6,166 PD per year are calculated from ambient exposure to the three pollutants, and the number of YLL ranges between 46,216 − 65,289 per year. PD and YLL are higher for males compared to females for most age groups except for 85 and over and infants under 1 year. An increase in PD and YLL is seen with age, except for infants under 1 year having the highest YLL. Israel experiences lower rates of PD and YLL compared to European nations with similar pollutant concentrations. This difference is largely attributed to Israel’s younger population. The findings underscore the importance of continued air quality improvements to protect public health in Israel.

**Graphical abstract:**

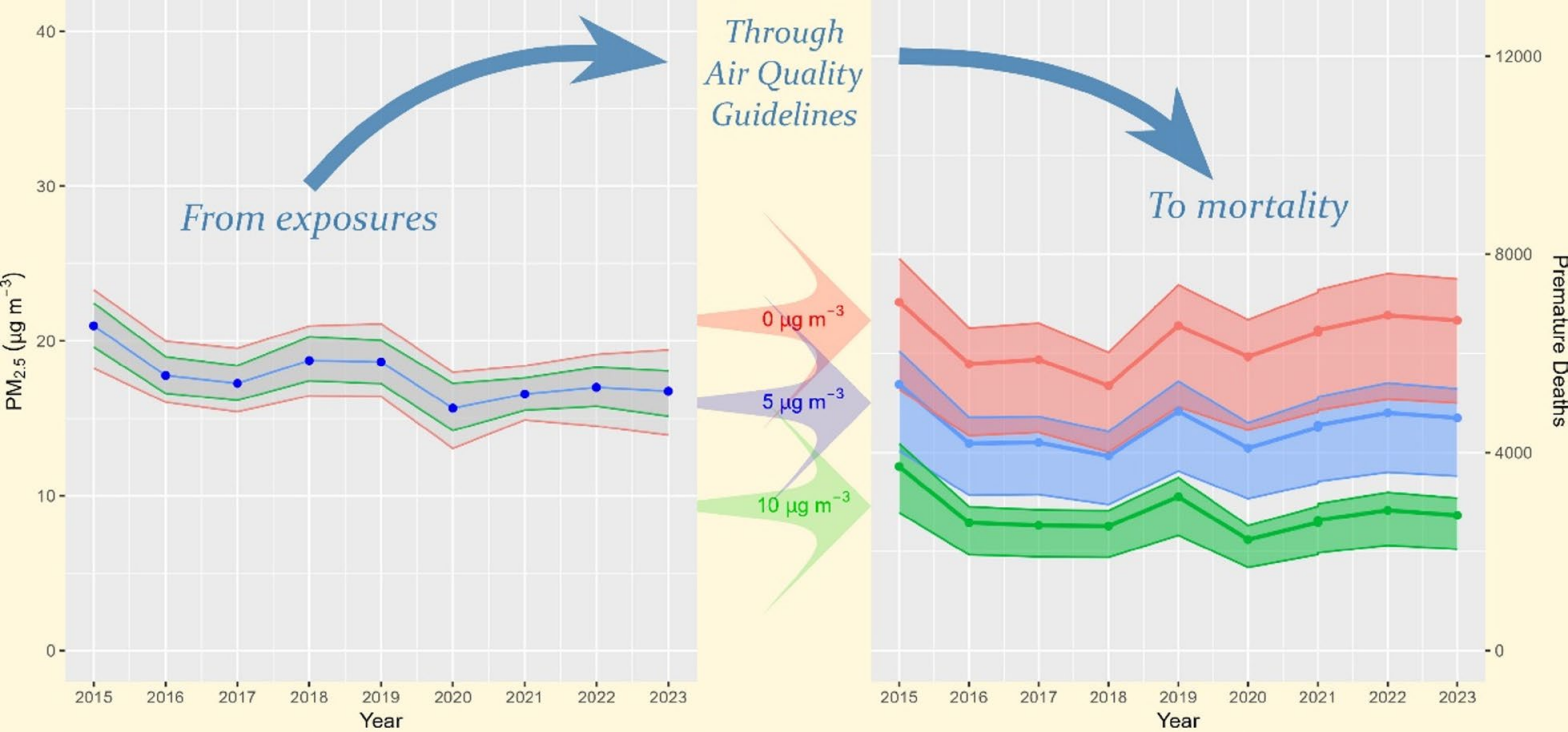

## Introduction

Ambient air pollution is a recognized global health concern [12]. The Global Burden of Disease Study data for 2021 (GBD) [10] estimates fine particulate matter, referring to particles with an aerodynamic diameter equal to or smaller than 2.5 μm (PM_2.5_) contributed globally 4.2 million premature deaths (PD), while ambient ozone (O_3_) contributed 837 thousands PD. Recognizing this significant public health burden, the World Health Organization (WHO) has established air quality guidelines (AQG) in 1987 (WHO [30]) to mitigate the adverse health impacts of major air pollutants, which were updated in 2006 [31]. In 2021, following a thorough literature review and evaluation of the most recent scientific evidence, an update was published to the AQG (WHO 2021) [33]. Among the pollutants for which AQG were established, PM_2.5_, nitrogen dioxide (NO_2_), and ozone (O_3_) are particularly harmful, linked, among others, to respiratory and cardiovascular diseases.

The health impacts of ambient air pollution are dependent on five parameters: exposure levels of the population, the mortality rate for the specific age and gender group, population size, the relative risk and the counter factual concentration. The relative risk is a measure that expresses the probability of premature death as a function of a fixed number of concentration units of air pollutant exposure. The counter factual concentration is the lowest exposure level of air pollutant below which there are no known health effects in the exposed population (WHO 2021) [33].

Several epidemiological studies in recent years have demonstrated that particulate air pollution may impact human health even at very low concentrations (e.g., [6, 25, 26]). In an analysis of 41 cohort studies, Burnett et al., [5] found that long-term exposure to PM_2.5_ is associated with mortality from noncommunicable diseases included lower respiratory illness with the lowest observed concentration of 2.4 µg/m^3^. These findings warrant the examination of a “no safe limit” scenario for PM_2.5_, with counterfactual concentration of 0 µg/m^3^.

In a recent report adopting the new WHO air quality guidelines for long-term exposures to PM_2.5_ and NO_2_ and short term exposures to O_3_, the authors examine the health risks related to ambient pollution levels in each of 27 Member States of the European Union and for additional 14 European countries [28]. The results show the main cause for premature deaths (PD) and years of life lost (YLL) to be PM_2.5_, which has the largest impact in central and eastern European countries, reaching as high as 241 PD per 100,000 inhabitants, and the lowest in Scandinavian countries, with less than 17 PD per 100,000 inhabitants.

Multiple studies examined the impact of ambient air pollution on public health in Israel in recent years (e.g., Berezovsky et al., 2024; [8]; Cohen et al., 2018 [7]; [19, 23, 29]). Two earlier estimates of PD from ambient PM_2.5_ in Israel were published in the past. Ginsberg et al., [11] used a WHO spread-sheet model and two additional models for cause-specific relative risks to estimate mortality rates from PM_2.5_ exposures in 2015. Results from the three models were 1,609, 1,908 and 2,253 PD. The OECD (OECD 2019) [24] published its latest estimate of PD from ambient PM_2.5_ in Israel for 2019. An estimate of 26.8 cases per 100,000 residents resulted in 2,280 PD. This estimate fitted the range published earlier by Ginsberg et al., [11].

This study examines the impact of these three pollutants on PD and YLL in Israel between 2015 and 2023. The analysis incorporates the most recent WHO air quality guidelines publication (WHO 2021) [33] for counterfactual concentrations and concentration-response functions. In addition, we use the Israeli Central Bureau of Statistics’ (ICBS) most updated data available about the population’s spatial distribution stratified by age and gender at the finest available census tract resolution for each year as well as mortality rates and life expectancy data. All these provide an updated and comprehensive assessment of air pollution’s impact on the public in Israel in terms of PD and YLL. Beyond estimating the health burden, the analysis is intended to clarify how these results can inform plausible policy action across emission-related domains and health-system planning under demographic change.

## Methodology

This study adopted the methodology in [28], using the finest resolution of demographic and exposure data available as described below.

### Exposure estimates

Exposure metrics to PM_2.5_, NO_2_ and O_3_ at the census tract resolution were calculated for each year between 2015 and 2023, including a total of approximately 2,760 populated tracts per year. Annual average concentrations of PM_2.5_ and NO_2_ were obtained from the Israeli Ministry of Environmental Protection’s air quality hybrid model. A detailed description of the model is given in Levy et al., [21]. The model incorporates annual means of hourly predictions from a photochemical model with annual means of in-situ measurements from a network of monitoring stations across the country, providing a comprehensive representation of air pollution levels. The model was recently extended to include SOMO35, which is the annual sum of daily maximum running 8-hours average concentrations of O_3_ above 35 ppb, divided by the number of days in a year, as the metric for short term exposure to O_3_. The Mann-Kendall test was performed on each of the exposure metrics time series between 2015 and 2023 to detect monotonic trends.

### Population, mortality and life expectancy data

Detailed total population size, categorized by age and gender groups at the census tract resolution, was sourced from the Israeli Central Bureau of Statistics (ICBS) for each year from 2015 to 2022 (ICBS 2023) [14]. For 2023, the population data of 2022 was used. In addition, the national totals of deaths attributed to all natural causes for each year between 2015 and 2023, as well as mean life expectancy for the years 2018–2022, categorized by age and gender groups were obtained from the ICBS (personal communication with ICBS) (Table SI 1). Life expectancy data was provided at a single year age by gender and regrouped to the age groups in Table SI 1 by population size weighted mean.

### Concentration-response functions and counterfactual concentrations

The study employs concentration-response functions based on attributable risk estimates, to quantify the relationship between exposure to specific pollutants and PD. The functions and counterfactual concentrations for PM_2.5_, NO_2_, and O_3_ are based on WHO (2021) as detailed in Table 1.

### Attributable PD

The attribution of PD to air pollution is calculated by quantifying the increased risk of mortality associated with exposure to pollutant concentrations exceeding the WHO air quality guidelines as the counterfactual concentrations in Israel. Epidemiological studies with multipollutant models concluded that the effects of NO_2_ may represent an overestimate in the likely range of up to 30% (WHO 2013) [32]. Accounting for the potential overlap with the long-term effects of PM_2.5_, when summing the PD attributed to PM_2.5_ and NO_2_, 30% of the NO_2_ effect was reduced.

### Calculation of years of life lost

Years of life lost (YLL) represents the difference between the age at death and the expected life expectancy for that individual. By multiplying the number of PD in each age and gender group by the average remaining life expectancy for that group, the study calculates the total YLL attributable to each pollutant [28].

**Table 1:** Relative risk, counterfactual concentrations and relevant health outcomes for fine particulate matter, nitrogen dioxide and ozone (WHO 2021) [33]

Pollutant	Exposure metrics	Relative Risk per 10 µg/m^3^ (95% CI)	Counterfactual concentration (ug/m^3^)	Health outcome
PM_2.5_	Population weighted annual mean	1.08 (1.06–1.09)	5	All-cause (natural) mortality in ages over 30 years in total population
NO_2_	Population weighted annual mean	1.02 (1.01–1.04)	10	All-cause (natural) mortality in ages over 30 years in total population
O_3_	SOMO35	1.0043 (1.0034–1.0052)	70	All-cause (natural) mortality in all ages in total population

## Results

### Population exposure levels

**Fig. 1:**
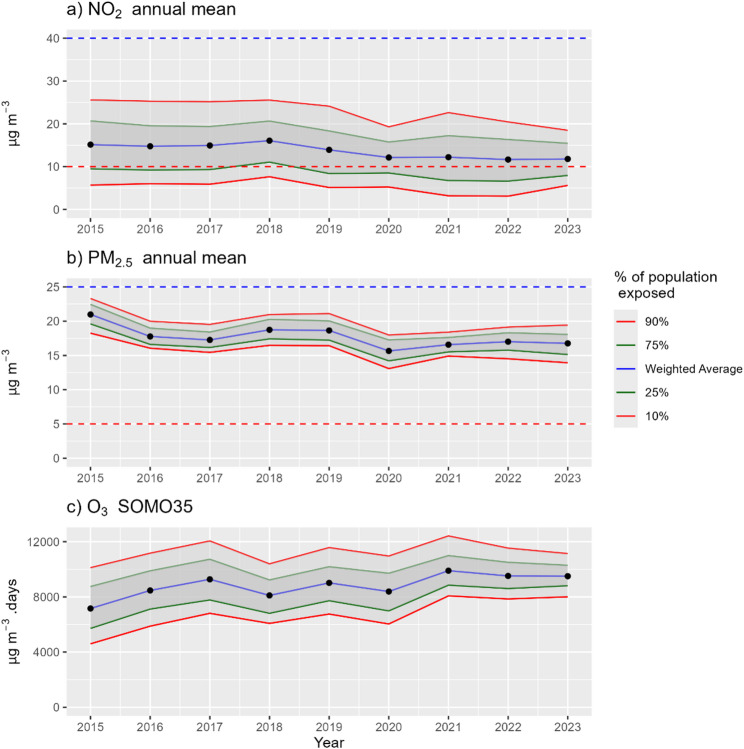
Population weighted average exposure concentrations (blue) and the concentration below which percents (10% and 90% in red, 25% and 75% in green) of population is exposed to, for annual mean of NO_2_ (**a**) and PM_2.5_ (**b**) and SOMO35 of O_3_ (**c**). Dashed red (blue) lines indicate national health (ambient) related Air Quality Guideline values

Time series of population weighted exposures to ambient concentrations are presented in Fig. 1. NO_2_ weighted concentrations exhibit a statistically significant decreasing trend between 2015 (15.1 µg/m^3^) and 2023 (11.8 µg/m^3^) (Kendall’s τ = −0.67, Z = −2.4, *p* < 0.05). This decrease is manifested in the percent of population exposed to ambient levels exceeding the national health AQ guideline for annual mean NO_2_ of 10 µg/m^3^, from 73% in 2015 to 63% in 2023. Weighted exposure to SOMO35 shows a statistically insignificant increase trend from 7,160 µg/m^3^. days to 9,502 µg/m^3^. days (Kendall’s τ = 0.5, Z = 1.8, *p* < 0.1). For PM_2.5_, no statistically significant trend is observed between 2015 and 2023 (Kendall’s τ = −0.5, Z = −1.8, *p* < 0.1).

### Premature death

**Fig. 2:**
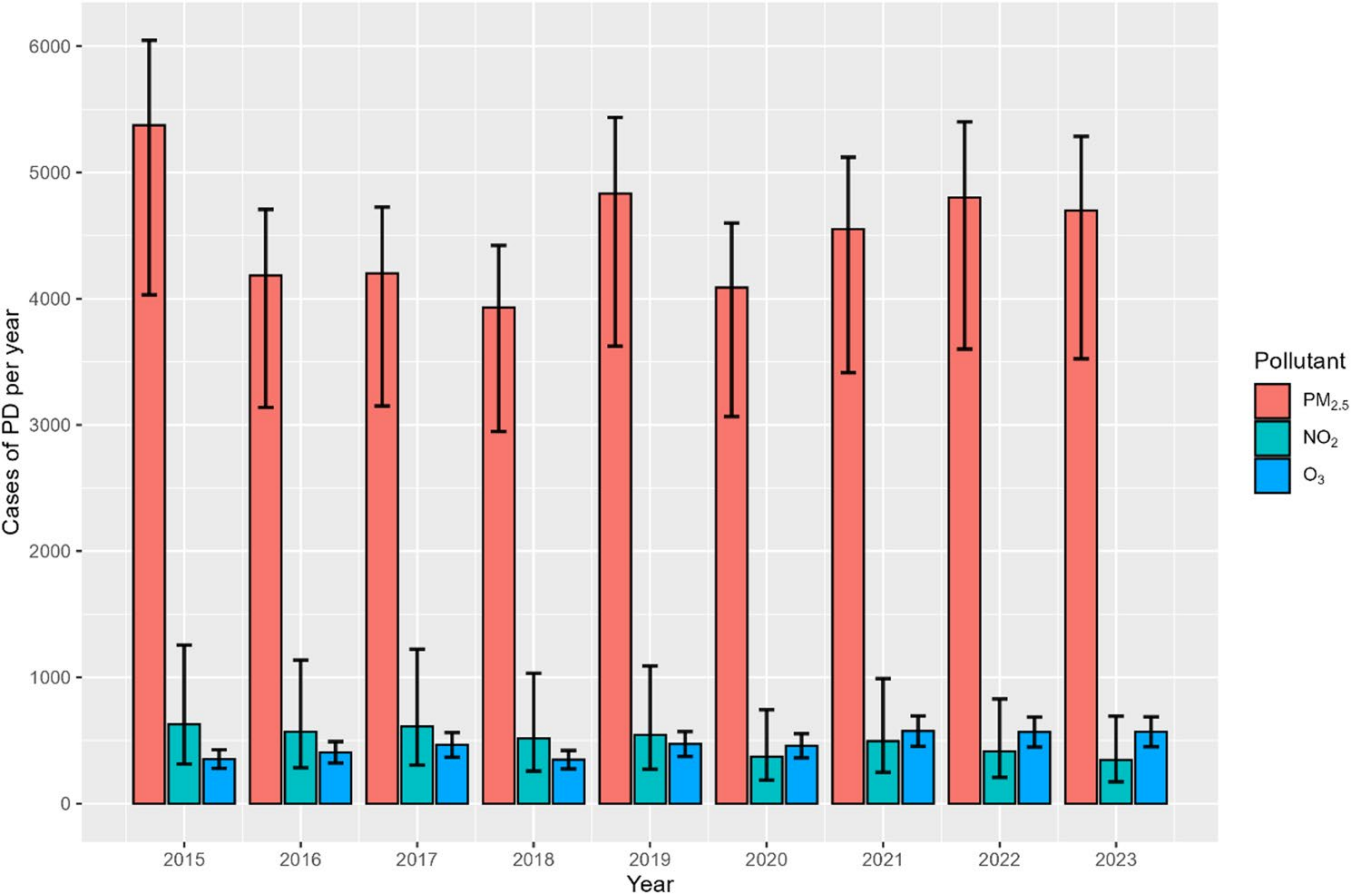
Annual cases of PD and 95% CI from each pollutant between 2015 and 2023. See Table 2 for data used in the figure

The analysis reveals a significant burden of PD in Israel attributable to long-term exposures to ambient PM_2.5_, NO_2_, and short term exposures to O_3_ (Fig. 2; Table 2). Between 2015 and 2023, the estimated sum of PD per year attributed to exposures to these three pollutants together ranged from 4,641(95% CI: 3,404-5,566) in 2018 to 6,166 (95% CI: 4,529-7,352) in 2015. Exposure to PM_2.5_ emerged as the most significant contributor to PD, accounting for the majority of estimated deaths each year. The highest number of PD attributed to PM_2.5_ (5,375, 95% CI: 4,031 − 6,046) occurred in 2015, a year marked by a high frequency of dust events that significantly elevated PM_2.5_ concentrations to a population weighted mean of 21.0 µg/m^3^ (Table 2).

While still a concern, NO_2_ exposure was associated with a lower number of PD compared to PM_2.5_, ranging between 346 (95% CI: 173–692) in 2023 and 628 (95% CI: 314-1,256) in 2015. Exposure to O_3_ accounted for about the same number of PD per year as NO_2_, ranging between 352 (95% CI: 278–426) in 2015, and 575 (95% CI: 454–695) in 2021.

### Years of life lost

Like premature death, YLL attributable to air pollution in Israel is substantial. Between 2015 and 2023, the estimated sum of YLL per year from all three pollutants together ranged from 55,893 (95% CI: 41,113 − 66,962) in 2018 to 76,458 (95% CI: 56,329 − 90,952) in 2015 (Table 3). Exposure to PM_2.5_ accounted for the largest proportion of YLL, ranging from 46,216 (95% CI: 34,662 − 51,993) in 2018 to 65,289 (95% CI: 48,966 − 73,450) in 2015. This is followed by YLL from chronic exposures to NO_2_ between 3,802 (95% CI: 1,902-7,605) YLL in 2023 and 7,218 (95% CI: 3,609 − 14,435) YLL in 2015. Acute exposures to O_3_ accounted for 5,547 (95% CI: 4,386-6,708) YLL in 2018.

Notably, O_3_ exposure was found to have a high impact on infants (under one year of age). This vulnerability stems from the fact that O_3_-related mortality is calculated from birth, unlike PM_2.5_ and NO_2_, for which mortality is only calculated from the age of 30. Even a small number of premature deaths in infancy can translate to a considerable loss in YLL due to the significant remaining life expectancy.

### Age and gender

**Fig. 3:**
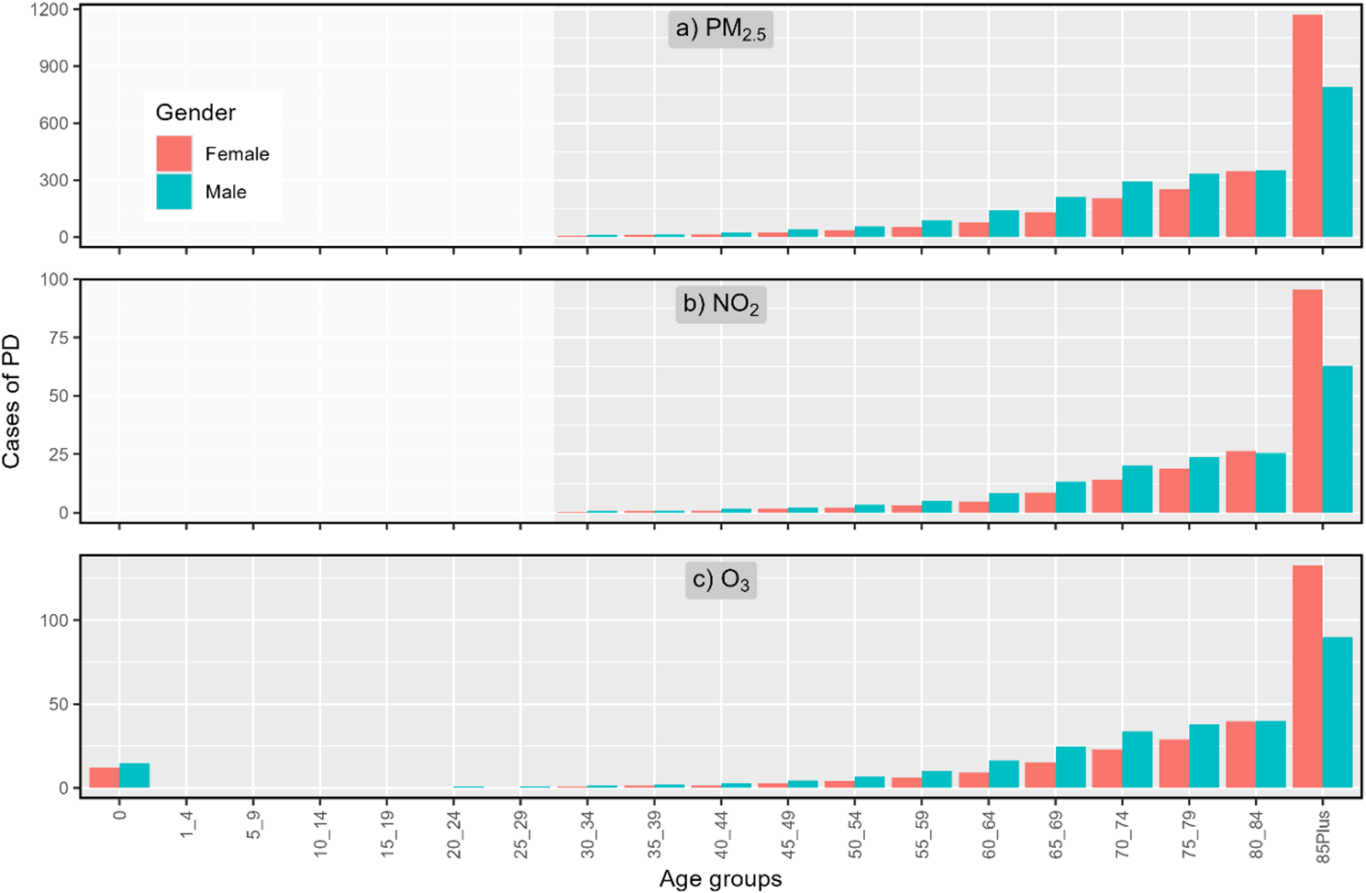
PD per age group and gender from exposure to PM_2.5_ (top), NO_2_ (center) and O_3_ (bottom) for the year 2023. Note the different scales in the y-axes. See Table SI 2 for data used in the figure

Figure 3 presents the PD by age and gender groups from exposures to each pollutant for the year 2023. While PD from exposures to PM_2.5_ and NO_2_ start only from age 30, PD from exposures to O_3_ starts from infancy. For almost all age groups except 85-plus, PD are higher for males than for females. However, when examining the normalized PD per 100,000 residents (Figure SI 1), PD among male is shown to be higher at all age groups, including the 85-plus group.

**Fig. 4:**
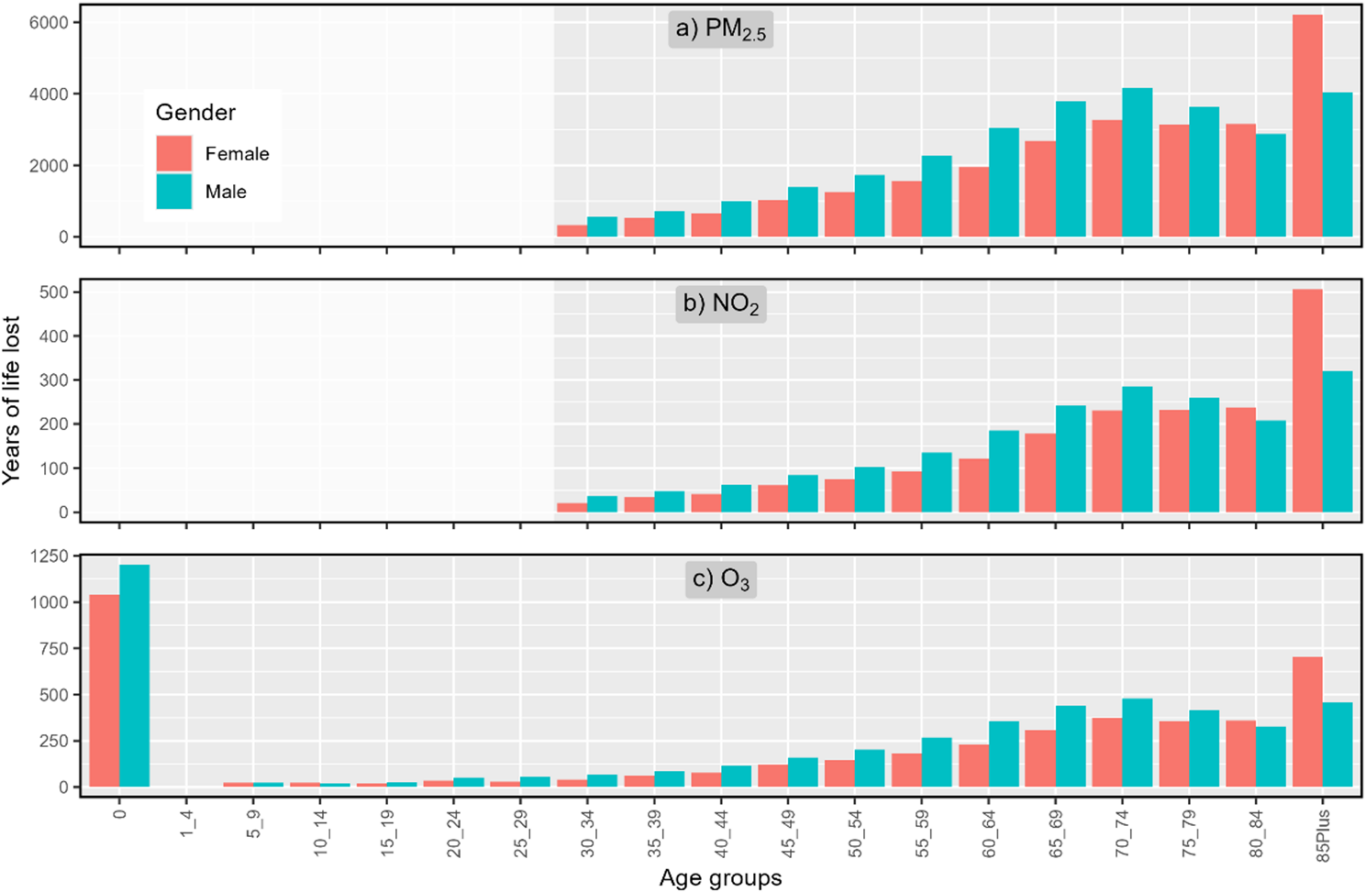
YLL per age group and gender from exposure to PM_2.5_ (top), NO_2_ (center) and O_3_ (bottom) for the year 2023. Note the different scales in the y-axes. See Table SI 3 for data used in the figure

A different trend is seen for YLL (Fig. 4), with an increase to the age group of 70–74, a decrease to the age group of 80–84, and a peak in the oldest age group of 85-plus. For O_3_, the PD in infancy (under 1) results in the highest YLL to be from this age group.

**Table 2:** Total population, population weighted exposure, PD with 95% CI and PD per 100k residents from PM2.5, NO2 and SOMO35 and the combined PD from three pollutants between 2015 and 2023

Year	Population (thousands)	PM_2.5_ annual mean (ug/m^3^)	PM_2.5_ PD (95% CI)	PM_2.5_ PD/100k population	NO_2_ annual mean (ug/m^3^)	PD from NO_2_ (95% CI)	NO_2_ PD/100k population	SOMO35 annual mean (ug/m^3^)	PD from SOMO35 (95% CI)	SOMO35 PD/100k population	Combined PD from three pollutants (95%, CI)
2015	8,397	21.0	5,375 (4,031 − 6,046)	64.0	15.1	628 (314-1,256)	7.5	7,160	352 (278–426)	4.2	6,166 (4,529-7,352)
2016	8,559	17.8	4,184 (3,138-4,707)	48.9	14.8	568 (284-1,137)	6.6	8,466	406 (321–492)	4.7	4,988 (3,658-5,994)
2017	8,725	17.3	4,201 (3,150-4,726)	48.1	14.9	611 (306-1,223)	7.0	9,275	465 (368–563)	5.3	5,094 (3,733-6,145)
2018	8,900	18.7	3,931 (2,948-4,422)	44.2	14.2	516 (258-1,033)	5.8	8,107	348 (275–421)	3.9	4,641 (3,404-5,566)
2019	9,070	18.6	4,832 (3,624-5,436)	53.3	13.9	545 (272-1,090)	6.0	9,015	472 (373–571)	5.2	5,686 (4,188-6,770)
2020	9,214	15.7	4,088 (3,066 − 4,599)	44.4	12.1	372 (186–744)	4.0	8,390	458 (362–554)	5.0	4,807 (3,559-5,674)
2021	9,378	16.6	4,552 (3,414-5,121)	48.5	12.2	495 (248–991)	5.3	9,899	575 (454–695)	6.1	5,473 (4,041 − 6,509)
2022	9,584	17.0	4,800 (3,600-5,401)	50.1	11.7	415 (207–829)	4.3	9,519	567 (448–686)	5.9	5,658 (4,194-6,667)
2023	^*^9,584	16.8	4,699 (3,524-5,286)	49.0	11.8	346 (173–692)	3.6	9,502	569 (450–688)	5.9	5,510 (4,095 − 6,459)

**Table 3:** Total population, population weighted exposure, YLL with 95% CI and PD per 100k residents from PM2.5, NO2 and SOMO35 and the combined YLL from three pollutants between 2015 and 2023

Year	Population (thousands)	PM_2.5_ annual mean (ug/m^3^)	PM_2.5_ YLL (95% CI)	PM_2.5_ YLL/100k population	NO_2_ annual mean (ug/m^3^)	YLL from NO_2_ (95% CI)	NO_2_ YLL/100k population	SOMO35 annual mean (ug/m^3^. days)	YLL from SOMO35 (95% CI)	SOMO35 YLL/100k population	Combined YLL from three pollutants (95%, CI)
2015	8,397	21.0	65,289 (48,966 − 73,450)	778	15.1	7,218 (3,609 − 14,435)	86.0	7,160	6,117 (4,837-7,397)	72.9	76,458 (56,329 − 90,952)
2016	8,559	17.8	50,816 (38,112 − 57,169)	594	14.8	6,512 (3,256 − 13,024)	76.1	8,466	7,078 (5,597-8,559)	82.7	62,453 (45,988 − 74,845)
2017	8,725	17.3	50,567 (37,925 − 56,888)	580	14.9	6,957 (3,479 − 13,914)	79.7	9,275	7,900 (6,247-9,554)	90.5	63,337 (46,607 − 76,182)
2018	8,900	18.7	46,216 (34,662 − 51,993)	519	14.2	5,902 (2,951 − 11,803)	66.3	8,107	5,547 (4,386-6,708)	62.3	55,893 (41,113 − 66,962)
2019	9,070	18.6	57,967 (43,476 − 65,213)	639	13.9	6,184 (3,092 − 12,367)	68.2	9,015	7,846 (6,204-9,489)	86.5	70,142 (51,844 − 83,359)
2020	9,214	15.7	48,546 (36,409 − 54,614)	527	12.1	4,193 (2,096 − 8,386)	45.5	8,390	7,188 (5,684-8,693)	78.0	58,669 (43,561 − 69,177)
2021	9,378	16.6	54,413 (40,810 − 61,215)	580	12.2	5,589 (2,795 − 11,179)	59.6	9,899	9,200 (7,275 − 11,126)	98.1	67,526 (50,041–80,166)
2022	9,584	17.0	56,239 (42,179 − 63,269)	587	11.7	4,579 (2,289-9,158)	47.8	9,519	8,884 (7,025 − 10,744)	92.7	68,329 (50,807 − 80,423)
2023	*9,584	16.8	55,019 (41,264 − 61,896)	574	11.8	3,802 (1,901-7,605)	39.7	9,502	8,896 (7,034 − 10,758)	92.8	66,576 (49,629 − 77,977)

### Sensitivity to counterfactual concentrations

In addition to the counterfactual concentration of 5 µg/m^3^, a sensitivity analysis was done for PM_2.5_ with counterfactual concentrations of 10 µg/m^3^, representing the WHO’s former air quality guidelines from 2013 [13] and 0 µg/m^3^ representing “no safe limit” approach. Results in Table 4 show PD cases range between 5,350 and 7,034 cases for counterfactual concentration of 0 µg/m^3^, indicating additional 1,419-1,966 cases per year compared to a range of 3,931-5,375 cases for counterfactual concentration of 5 µg/m^3^. In contrast, for counterfactual concentration of 10 µg/m^3^ there are between 2,224 and 3,710 cases, indicating fewer 1,419-1,966 cases per year compared to the number of YLL for a counterfactual concentration of 5 µg/m^3^.

**Table 4:** PD cases for three counterfactual concentrations (0, 5 & 10 µg/m3), and the addition (reduction) in PD when reducing (increasing) the counterfactual concentration to 0 (10) µg/m3

Year	Counterfactual concentration			Difference	
	0 µg/m^3^	5 µg/m^3^	10 µg/m^3^	5->0 µg/m^3^	5->10 µg/m^3^
2015	7,034	5,375	3,715	1,660	−1,660
2016	5,786	4,184	2,582	1,602	−1,602
2017	5,875	4,201	2,527	1,674	−1,674
2018	5,350	3,931	2,512	1,419	−1,419
2019	6,560	4,832	3,104	1,728	−1,728
2020	5,934	4,088	2,243	1,846	−1,846
2021	6,471	4,552	2,633	1,919	−1,919
2022	6,767	4,800	2,834	1,966	−1,966
2023	6,665	4,699	2,732	1,966	−1,966

### Comparison with European countries

To contextualize the findings within a broader geographical context, the study compares the rates of PD and YLL in Israel to those reported for 41 European countries in Soares et al., [28]. This comparison in considers data from 2020, the most recent year for which comprehensive data was available for both Israel and the European countries.

**Fig. 5:**
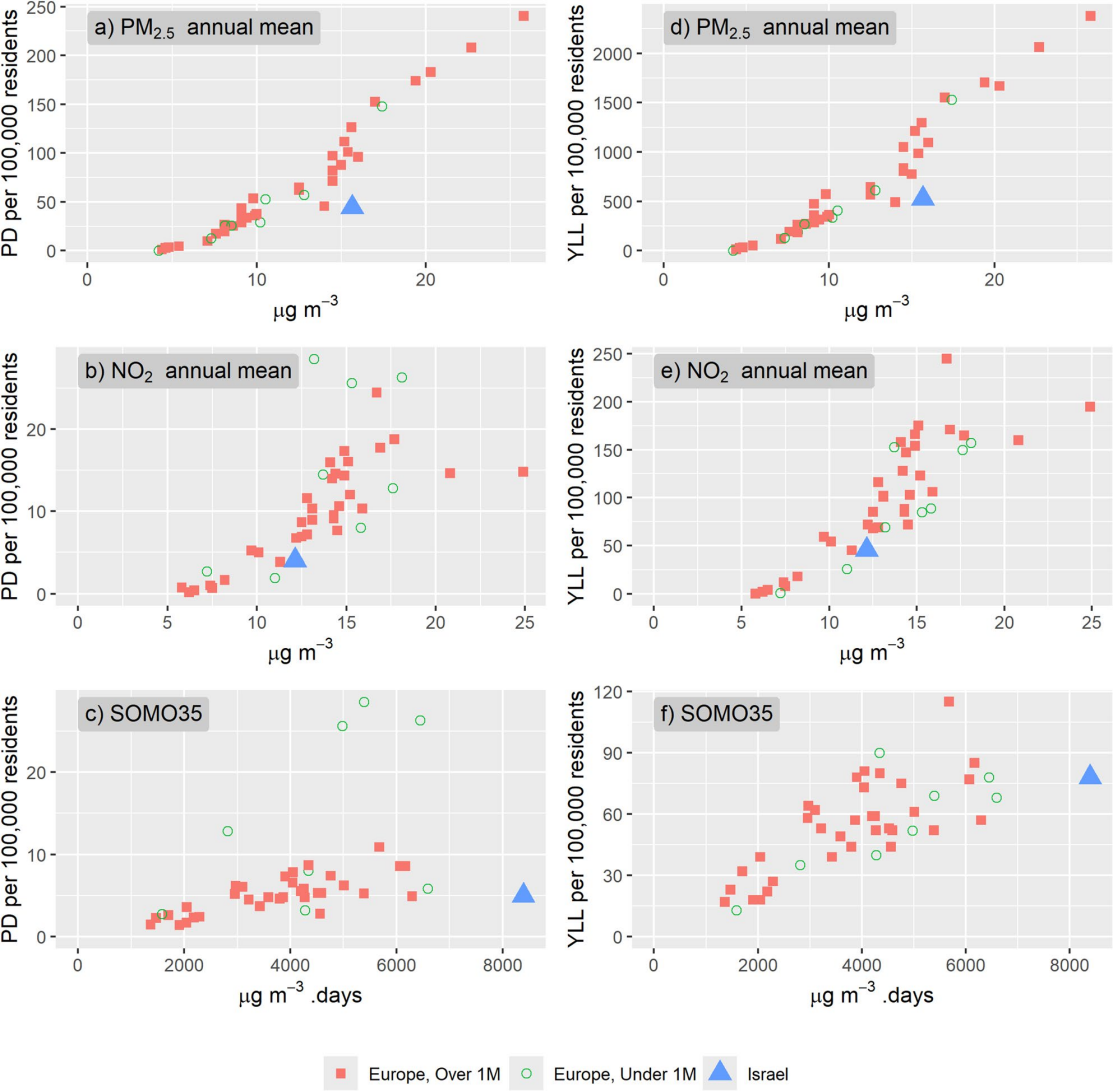
PD (left) and YLL (right) per 100,000 residents from exposures to PM_2.5_ (top), NO_2_ (middle) and O_3_ (bottom) in 2020 for european countris (red squares) and Israel (blie triangle). European countries with population under 1 million residents are in green circle. See Table SI 4 for data used in the figure

Despite the concerning levels of air pollution and related mortality in Israel, the study revealed that the rates of PD and YLL per 100,000 residents in Israel were consistently lower compared to European countries with similar pollutant concentrations (Table SI 4 and Fig. 5). For instance, in 2020, the population weighted annual mean PM_2.5_ in Isarel was 15.7 µg/m^3^, and the PD rate attributed to PM_2.5_ was 44.4 deaths per 100,000 residents. This mortality rate is significantly lower than rates observed in Slovakia, Greece, and Italy with slightly lower weighted exposures to PM_2.5_ of 14.5, 14.5 and 15.0 µg/m^3^_,_ respectively, and 71, 82 and 88 deaths per 100,000, respectively. Similarly, this difference is also manifested in YLL, with 527 YLL per 100,000 residents in Israel, compared to 838, 804 and 775 in Slovakia, Greece and Italy, respectively.

With the relative risk being the same for all age and gender groups, the number of PD cases in each age-gender group is the result of the absolute number of individuals in each group on the one hand, and the mortality rates for that group, on the other. With mortality rates for all age groups being higher for male than for female, even though the absolute PD cases are higher among females at the ages of 85-plus, the normalized PD at that age group are higher among male.

The lower PD rates per 100,000 residents in Israel compared to European countries is also manifested for NO_2_, with a population weighted mean of 12.1 and 4.0 PD per 100,000 residents in Israel, compared to the NO_2_ weighted means in Czechia (12.5 µg/m^3^), France (12.2 µg/m^3^) and Portugal (12.5 µg/m^3^) and 6.9, 6.8 and 8.7, respectively, PD per 100,000 residents.

For O_3_, the population’s exposure in Israel in 2020 (8,390 µg/m^3^. days, Fig. 5 and Table SI 4) is much higher compared to all other European countries reported in Soares et al., [28], with the highest being 6,592 µg/m^3^. days in Malta. Yet, the PD per 100,000 residents from O_3_ exposures in Israel (5.0) is well within the range reported for other European countries (from 1.4 to 8.6, Fig. 5, Table SI 4), and is lower compared to some Southern European countries with the highest reported insolation like Greece, Malta and Italy (8.6, 5.8 and 8.6, respectively), and even some Central European countries like Austria and Czechia (5.3 and 5.8 PD per 100,000, respectively) with a colder climate.

**Fig. 6:**
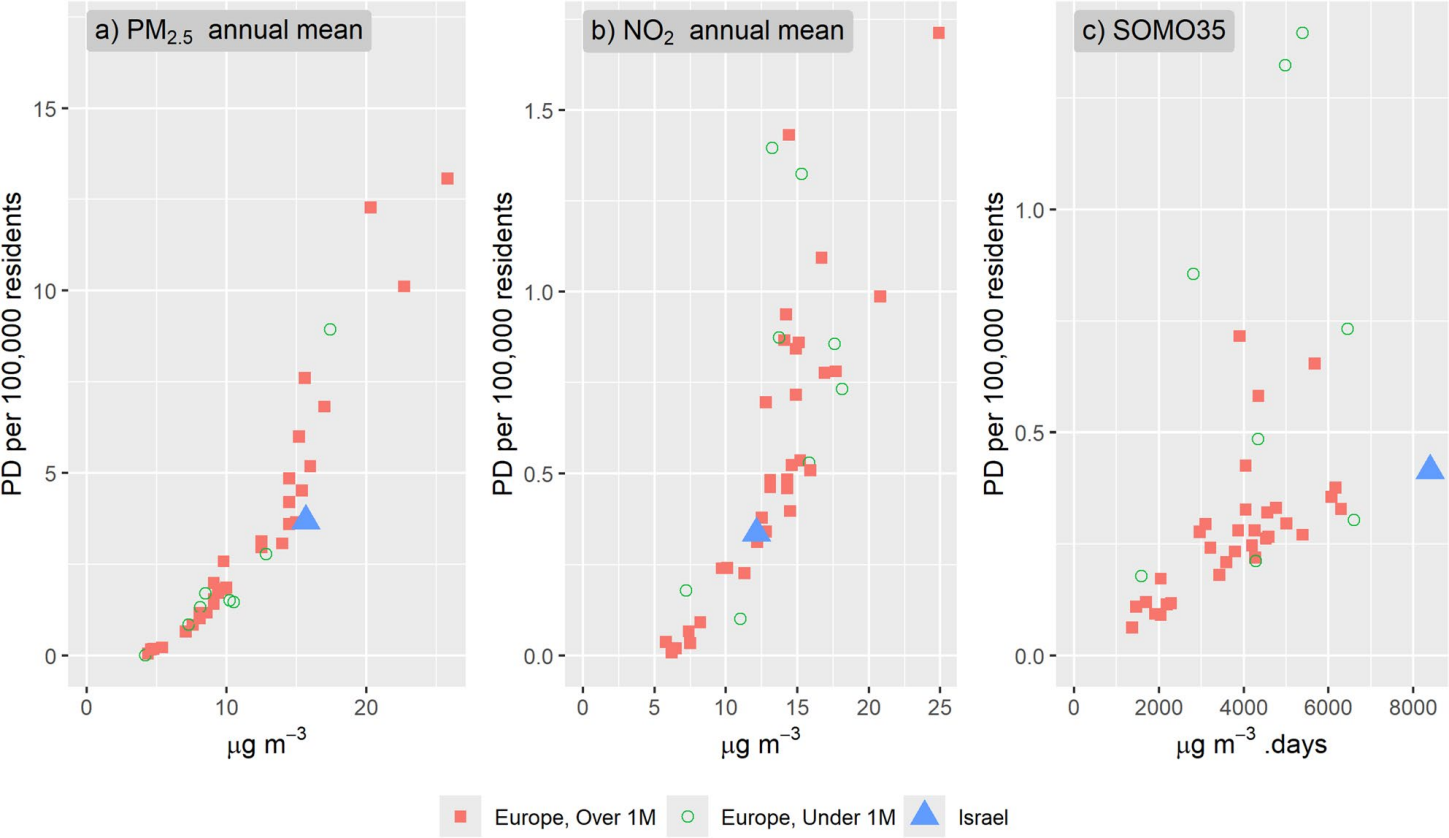
PD per 100,000 residents for population over 65 years only, from exposures to PM_2.5_, NO_2_ and O_3_ in 2020 for european countris (red squares) and Israel (blie triangle). European countries with population under 1 million residents are in green circle. See Table SI 4 for data used in the figure

This difference in PD rates between Israel and European countries is primarily attributed to the age distribution of the populations. According to World Bank data (World Bank Data 2024) [34], in 2022 Israel had 12.0% of the population aged over 65, a smaller proportion compared to all European countries, except Türkiye (8.6%) and Kosovo (10.2%) (Figure SI 2a, Table SI 4). In addition, Israel had the highest percent of young population between the ages of 0–14, 28.1% (Figure SI 2b, Table SI 4). With the elder population being at elevated risk of PD from exposure to air pollution as discussed above, a smaller proportion of the population at older ages implies lower PD rates for a given level of exposure to air pollutants. To support this argument, Fig. 6 and Table SI 4 present the PD per 100,000 residents for the population over 65 years from each of the pollutants. For this age group, the PD rates in Israel (blue triangle) from PM_2.5_ (3.70 PD/100,000 at 15.7 µg/m^3^) is at the lower range compared to other countries with similar exposure levels like Croatia, Poland and Albania with 4.52, 5.18 and 7.59 PD/100,000, respectively at 15.4, 16.0 and 15.6 µg/m^3^, respectively. For NO_2_, PD rates for ages over 65 are well within the observed PD rates in other countries. For O_3_ (SOMO35), even though the exposure levels in Israel are much higher, the PD rate for the elder population (0.41 PD per 100,000) is not the highest, yet it continues the extrapolated trend of the ration between PD per 100,000 residents and exposure.

## Discussion

This study quantifies PD and YLL from ambient exposures to three air pollutants (PM_2.5_, NO_2_ and O_3_) in Israel for each year between 2015 and 2023. The study uses the finest available resolution data at the census tract level for both population, further separated by gender and age groups and exposure estimates.

The combined effect of exposures to the three pollutants ranges from 4,641(95% CI: 3,404-5,566) PD in 2018 to 6,166 (95% CI: 4,529-7,352) PD in 2015, and from 55,893 (95% CI: 41,113 − 66,962) YLL in 2018 to 76,458 (95% CI: 56,329 − 90,952) YLL in 2015.

The main impact on PD is from exposure to PM_2.5_, ranging between 3,931 and 5,375 PD per year. These results are higher compared to earlier estimates for PD from PM_2.5_ in Israel which ranged between 1,609 and 2,253 for 2015 and 2,280 for 2019. The main reason for the increase in PD between this study and earlier estimates is the use of the WHO’s new air quality guidelines for PM_2.5_ from 2021 (WHO 2021) [33] as the counterfactual concentrations. Our results show that a change of 5 µg/m^3^ in the counterfactual concentrations for PM_2.5_ from the current WHO’s air quality guideline of 5 µg/m^3^ to the former air quality guideline of 10 µg/m^3^ or to a “no safe limit” of 0 µg/m^3^, will result in a lower (an additional) number of PD cases of between 1,419 and 1,966 per year.

The current dose-response functions to PM_2.5_ are derived exclusively from populations aged 30 and above. Excluding younger populations, many of whom belong to vulnerable groups, from these calculations leaves them inadequately assessed in terms of their long-term health impacts, particularly regarding outcomes such as mortality. Moreover, sex-based physiological and biological differences necessitate a more nuanced approach to evaluating health risks. These disparities highlight the importance of integrating sex-specific considerations into preventive and therapeutic strategies, particularly in efforts aimed at reducing adverse outcomes such as mortality among women.

When comparing the results presented in this study to similar calculation done for 41 European countries, both PD and YLL per 100,000 residents are lower in Israel than in countries with the same levels of ambient pollution levels. This is attributed to the lower proportion of the elderly population in Israel.

### Policy-relevance pathways

Taken together, the results help clarify where policy leverage is most plausibly linked to health gains. First, the dominance of PM2.5 in PD and YLL points to emission-related policy arenas that influence near-population exposures—transport systems and urban planning, stationary combustion and energy production, and enforcement and governance relevant to diffuse sources such as open waste burning. Second, the contrasting trends of decreasing NO_2_ and increasing O_3_ highlight the relevance of integrated, multi-pollutant management when precursor emissions change over time, to avoid unintended co-pollutant tradeoffs and to manage seasonal/high-exposure episodes. Third, the lower crude burden compared to Europe is partly demographic; as Israel’s population ages, the attributable burden under similar ambient concentrations is likely to increase, strengthening the relevance of preventive air-quality action and preparedness in health and social-care settings.

The emissions inventory for Israel, published by the Israeli Ministry of Environmental Protection (IMoEP) shows that the three major contributing sectors to local emissions of PM_2.5_ in 2024 were municipal waste burning (20%), on-road traffic (19%) and wildfires (18%) (IMoEP 2025) [18]. For NO_x_, the major contributing sectors are power production (34%), on-road traffic (17%) and industry (15%). Although these sources account only for the local (and mostly anthropogenic) contributions and do not include transboundary and long-range transport of anthropogenic nor natural sources (local or remote) of these pollutants, or the production of secondary particles, they provide some insight into the diversity of emissions sources impacting ambient concentrations. This source profile helps indicate which emission-related policy domains are most plausibly connected to near-term reductions in PM_2.5_ and NO_x_, while recognizing that ambient concentrations also reflect secondary formation and regional transport.

The main cause for the lack of a temporal trend in PM_2.5_ is assumed to be the large contribution of natural dust to particulate matter concentrations in the region (Belachsen and Broday 2024) [2], which was shown to have a wide variability from year to year [9].

A statistically significant decreasing trend in exposure to NO_2_ concentrations and associated mortality over the study period is observed, attributed mostly to reductions in vehicle, power generation and industrial emissions (IMoEP 2024) [17] due to improvements in technologies and/or policy measures, but also because of other forcings like the COVID pandemic in 2020 and 2021.

Recent epidemiological studies have demonstrated the significant impact of NO_2_ exposures on population health, with clear evidence of their contribution to health outcomes. However, relatively partial data are available regarding their association with severe health outcomes such as mortality. Moreover, the dose-response functions commonly used in these assessments are based primarily on adult populations over the age of 30, which hinders comprehensive and evidence-based evaluations that reflect the broader population, including younger and potentially more vulnerable groups.

Exposure to O_3_ presented a contrasting trend over time compared to NO_2_, with both exposure and PD increasing over the same period. The increase in O_3_ is attributed to the decrease in the emissions of nitrogen oxides, reducing O_3_ titration in nitrogen-rich regions like urban centers, and allowing O_3_ to be closer to background levels, though still lower [20].

Recent changes in ambient O_3_ concentrations across urban areas indicate a growing risk of exposure, primarily due to increasing population density and the expanding scale of urban settlements. Currently, demographic trends show that the urban population comprises the majority of Israel’s total population, underscoring the potential for widespread exposure and the relevance of exposure-management in densely populated region (e.g., risk communication during high-O_3_ periods and protection of clinically vulnerable groups).

## Limitations

There are several limitations to the study that may contribute to an underestimation of the full impact of air pollution on premature death in Israel. The study doesn’t account for the combined effect of the three pollutants considered, which may have an aggravating impact compared to the individual effect of each pollutant. For PM_2.5_, quantified by the sum of mass of all particles smaller than 2.5 micrometer, the measure is biased to larger particles, while recent studies [4, 27] suggest that the smaller particles, and particularly ultrafine particle (smaller than 0.1 micrometer), and the composition of the particles might be a significant factor in their potential health impact.

We also didn’t account for the impact of other pollutants that are known to be harmful to human health, like VOC’s (such as benzene and formaldehyde) and heavy metals. For these pollutants, exposure levels for the entire population were not available.

Estimates of PD from PM_2.5_ in this study do not account for the full impact of ambient air pollution on public health and young population in particular, as they do not account for other effects like morbidity and miscarriages.

The relative risks of the pollutants were adopted from the WHO’s guidelines, and are not based on the Israeli population. As there may be heterogeneities in the associations between ambient pollution and health outcomes (Abu Ahmad et al. 2024) [1], a population-specific relative risk functions for each of the pollutants considered might give a more accurate estimate of the health impact of air pollution in Israel,

Last, the study also doesn’t account for the full impact of air pollution in Israel, as it doesn’t calculate morbidity, the economic costs of YLL, hospitalizations or loss of workdays.

### Policy Implications

The findings point to three policy-relevant domains: reducing PM_2.5_-dominated near-population emissions, managing emerging O_3_-related risks through an integrated multi-pollutant lens, and planning for increasing vulnerability as the population ages.

To minimize the negative health impacts, policy action is most plausibly focused on reducing emissions near residential areas, particularly traffic-related PM_2.5_ and NO_2_ in major urban centers. A reduction in vehicle milage and related congestion may be achieved by developing effective public transport in various regions (e.g., intra- and inter-urban) of complementing types (e.g., trains, subways, light-trains and buses) and promoting low emissions vehicles (e.g., electric or hybrid). Another major emission source of air pollution in Israel is waste burning, with a contribution of 20% to PM_2.5_ emissions in 2024 (IMoEP 2025) [18]. Abating these emissions is more challenging because of the illegal nature of the activity and the fact that it involves a wider solution to waste management in Israel. Last, a transition of power production from fossil fuels to renewable energy sources, and in particular the conversion of the two coal-powered power plants operating in Israel, which are among the top single emitters of air pollutants and greenhouse gasses in Israel in terms of external costs (IMoEP 2025) [18], is a major lever for reducing emissions of both local air pollutants and greenhouse gases. The observed increase in O_3_-related PD suggests growing relevance for policy and regulatory arenas that address photochemical pollution. In NOx-rich urban environments, changes in NO_x_ can alter O_3_ through reduced titration, implying the importance of considering O_3_ and its precursors (NO_x_/VOCs) together rather than through single-pollutant targets alone. This also strengthens the case for seasonal exposure-management during high-O_3_ periods, especially for clinically vulnerable groups.

Ambient concentrations in Israel reflect a mix of (i) locally controllable anthropogenic emissions (e.g., traffic, industry, power generation, waste burning), (ii) regional/transboundary transport (including episodes originating from neighboring regions), and (iii) meteorology-driven natural dust transport. While Israel cannot prevent regional transport of dust, it can reduce peak exposures and health risks through (a) local emission abatement measures like temporary emission curtailment where feasible — especially during stagnation/thermal inversion conditions when local sources dominate near-ground concentrations —and (b) exposure-management measures during forecasted dust episodes (alerts to the public, targeted protection of vulnerable groups and indoor air filtration in sensitive institutions). Because Israel’s population is aging, a larger share of the attributable burden is expected to concentrate in older and clinically vulnerable populations. This increases the relevance of preparedness and exposure-reduction measures in health and social-care settings (e.g., long-term care and hospitals), alongside targeted risk communication during pollution episodes.

These policy-relevant domains align with existing regulatory mechanisms in Israel. The Clean Air Law (CAL) in Israel (IMoEP 2008) [15] provides the main regulatory framework for reducing ambient air pollution through three complementary levers: (1) periodically reviewed and updated Air Quality Standards (including environmental, target and alert values), (2) enforceable emission permits for major sources requiring best available techniques, and (3) recurring National Plans that prioritize high-impact sectors and hotspots and define time-bound measures. Evaluations of CAL implementation suggest that the health and externality benefits of emission reduction measures outweigh compliance costs (IMoEP 2018) [16]. Observed long-term declines in NO_x_ concentrations are consistent with technological improvements and strengthened emission controls, particularly in the transport sector [22].

## Conclusions

The economic costs of air pollution and greenhouse gases emissions in Israel for the year 2024 were recently estimated to be 37 Billion NIS, of which 20.2 Billion are attributed to local air pollutants and 16.7 Billion to greenhouse gases (IMoEP 2025) [18]. These estimates, together with the health effects presented here, emphasize the significant impact of air pollution in Israel, with both economic and public health implications. In spite of the decreasing trend in both emissions (as reported in IMoEP (2025) [18]) and mortality, the estimates presented here underscore the continued policy relevance of reducing controllable emissions and managing exposure peaks, alongside longer-term planning under demographic change. PM_2.5_ is the most substantial air pollutant contributing to PD and YLL in Israel, highlighting the need for targeted interventions to reduce emissions from key sources. The high contribution of natural sources to ambient PM in Israel only accentuates the need to reduce anthropogenic emissions to maintain acceptable exposure levels to this pollutant.

The increasing O_3_-related burden highlights the importance of integrated, multi-pollutant approaches and seasonal exposure management during high-O_3_ periods, particularly for clinically vulnerable populations.

While Israel experiences lower crude rates of premature mortality and YLL per capita compared to other European countries with similar pollution levels, this difference is largely attributable to its younger population. The aging of the Israeli population underscores the need for proactive measures to improve air quality between different ministries with related laws and protect public health in the coming years.

The adoption of updated methodology and incorporating the latest scientific evidence, has resulted in higher and more accurate estimates of air pollution’s premature death estimations in Israel. This underscores the importance of using the most current data and methodologies when assessing and addressing the health impacts of environmental hazards.

Overall, the findings indicate that PM_2.5_ remains the primary driver of PD and YLL, that O_3_-related risks are becoming more salient as emission profiles change, and that demographic aging is likely to increase the attributable burden—together clarifying the policy arenas in which action could reasonably occur without assigning responsibility to specific agencies.

## Supplementary Information

Supplementary Material 1

## Data Availability

No datasets were generated or analysed during the current study.
