# Supplementary material for: Premature mortality and years of life lost attributable to ambient air pollution in Israel, compared to Europe: analysis and implications
Source: Isr J Health Policy Res. 2026 Mar 9;15:7. doi: 10.1186/s13584-026-00753-4 (PMC12969856; doi:10.1186/s13584-026-00753-4)
Supplement: Supplementary file 1 — Supplementary Material 1 [file 13584_2026_753_MOESM1_ESM.docx]

Supplementary Information

Table SI1: Life expectancy between 2018-2022 and mortality rates per 100,000 residents for every year from 2015 and 2022 by age groups and gender (Source: ICBS, personal communication, 2024).

Table SI2: Data used for Figure 3. PD per age group and gender from exposure to PM_2.5_, NO_2_ and O_3_ for the year 2023.

Table SI3: Data used for Figure 4. YLL per age group and gender from exposure to PM_2.5_, NO_2_ and O_3_ for the year 2023.

Table SI4: Total population size, percent of population over 65 years, and between 0-14 years, exposures, PD, PD per 100,000 residents, YLL and YLL per 100,000 residents for PM_2.5_, NO_2_ and SOMO35 for countries in Europe and Israel. Data for European countries taken from Soares et al. (2022).

Figure SI1: Normalized PD per 100,000 residents by age group and gender from exposure to PM_2.5_ (top), NO_2_ (center) and O_3_ (bottom) for the year 2023. Note the different scales in the y-axes.

Figure SI2: Percent of population over 65 years old (a) and population between ages 0-14 (b) for Israel (orange) and European countries. Source: World Bank data for 2022.

Table SI 1: Life expectancy between 2018-2022 and mortality rates per 100,000 residents for every year from 2015 and 2022 by age groups and gender (Source: ICBS, personal communication, 2024).

| Gender | Age group | Life expectancy  2018- 2022 | Mortality rate | | | | | | | |
| --- | --- | --- | --- | --- | --- | --- | --- | --- | --- | --- |
|  |  |  | 2015 | 2016 | 2017 | 2018 | 2019 | 2020 | 2021 | 2022 |
| Male | Up to 1 | 80.8 | 329 | 334 | 317 | 292 | 335 | 255 | 315 | 296 |
|  | 1-4 | 78.6 | 14 | 13 | 15 | 11 | 13 | 10 | 15 | 14 |
|  | 5-9 | 74.1 | 9 | 10 | 7 | 8 | 6 | 5 | 7 | 6 |
|  | 10-14 | 69.1 | 10 | 8 | 6 | 6 | 6 | 7 | 6 | 6 |
|  | 15-19 | 64.2 | 19 | 14 | 17 | 17 | 14 | 11 | 9 | 10 |
|  | 20-24 | 59.3 | 20 | 19 | 16 | 18 | 15 | 19 | 22 | 22 |
|  | 25-29 | 54.5 | 20 | 24 | 20 | 25 | 24 | 22 | 29 | 29 |
|  | 30-34 | 49.6 | 37 | 36 | 43 | 33 | 36 | 39 | 39 | 39 |
|  | 35-39 | 44.8 | 50 | 51 | 47 | 52 | 57 | 55 | 60 | 57 |
|  | 40-44 | 40.0 | 99 | 91 | 86 | 96 | 76 | 82 | 96 | 91 |
|  | 45-49 | 35.2 | 161 | 158 | 163 | 154 | 157 | 156 | 149 | 150 |
|  | 50-54 | 30.6 | 340 | 306 | 297 | 312 | 271 | 282 | 293 | 248 |
|  | 55-59 | 26.2 | 504 | 490 | 498 | 489 | 498 | 498 | 499 | 458 |
|  | 60-64 | 21.9 | 818 | 804 | 770 | 777 | 791 | 856 | 851 | 796 |
|  | 65-69 | 17.9 | 1,344 | 1,260 | 1,257 | 1,246 | 1,179 | 1,296 | 1,321 | 1,311 |
|  | 70-74 | 14.2 | 2,236 | 2,114 | 2,015 | 1,982 | 1,927 | 2,142 | 2,164 | 2,039 |
|  | 75-79 | 10.9 | 3,772 | 3,628 | 3,530 | 3,380 | 3,444 | 3,673 | 3,532 | 3,407 |
|  | 80-84 | 8.2 | 6,605 | 6,021 | 6,203 | 5,844 | 5,834 | 6,186 | 6,142 | 6,437 |
|  | 85< | 5.1 | 14,946 | 13,926 | 14,893 | 13,777 | 14,141 | 14,884 | 14,856 | 15,401 |
|  | All male |  | 509 | 484 | 495 | 480 | 484 | 518 | 526 | 529 |
| Female | Up to 1 | 84.8 | 295 | 283 | 277 | 290 | 239 | 210 | 240 | 260 |
|  | 1-4 | 82.6 | 11 | 11 | 14 | 10 | 14 | 9 | 12 | 14 |
|  | 5-9 | 78.1 | 5 | 7 | 7 | 5 | 4 | 6 | 4 | 6 |
|  | 10-14 | 73.1 | 9 | 9 | 4 | 5 | 7 | 6 | 6 | 7 |
|  | 15-19 | 68.1 | 8 | 10 | 10 | 8 | 8 | 8 | 9 | 8 |
|  | 20-24 | 63.2 | 10 | 11 | 12 | 8 | 12 | 11 | 13 | 14 |
|  | 25-29 | 58.3 | 14 | 15 | 15 | 15 | 15 | 15 | 14 | 14 |
|  | 30-34 | 53.3 | 20 | 26 | 25 | 19 | 21 | 20 | 24 | 22 |
|  | 35-39 | 48.4 | 33 | 48 | 36 | 37 | 40 | 37 | 35 | 39 |
|  | 40-44 | 43.5 | 67 | 71 | 66 | 58 | 62 | 61 | 63 | 55 |
|  | 45-49 | 38.7 | 106 | 103 | 95 | 96 | 89 | 95 | 108 | 99 |
|  | 50-54 | 33.9 | 200 | 172 | 160 | 174 | 172 | 165 | 161 | 157 |
|  | 55-59 | 29.3 | 285 | 288 | 285 | 266 | 268 | 266 | 264 | 267 |
|  | 60-64 | 24.7 | 458 | 471 | 467 | 437 | 449 | 453 | 442 | 410 |
|  | 65-69 | 20.3 | 797 | 740 | 757 | 735 | 749 | 708 | 753 | 718 |
|  | 70-74 | 16.1 | 1,345 | 1,275 | 1,230 | 1,201 | 1,204 | 1,211 | 1,305 | 1,194 |
|  | 75-79 | 12.3 | 2,522 | 2,383 | 2,334 | 2,266 | 2,295 | 2,241 | 2,226 | 2,113 |
|  | 80-84 | 9.1 | 5,014 | 4,969 | 4,524 | 4,450 | 4,413 | 4,571 | 4,663 | 4,601 |
|  | 85< | 5.3 | 13,182 | 13,295 | 12,855 | 12,718 | 13,198 | 13,657 | 13,821 | 14,016 |
|  | All female |  | 505 | 502 | 486 | 480 | 491 | 501 | 516 | 514 |

Table SI 2: Data used for Figure 3. PD per age group and gender from exposure to PM_2.5_, NO_2_ and O_3_ for the year 2023.

| Age group | PM_2.5_ PD cases | | NO_2_ PD cases | | O_3_ PD cases | |
| --- | --- | --- | --- | --- | --- | --- |
|  | Male | Female | Male | Female | Male | Female |
| 0-1 |  |  |  |  | 15 | 12 |
| 1-4 |  |  |  |  | 0 | 0 |
| 5-9 |  |  |  |  | 0 | 0 |
| 10-14 |  |  |  |  | 0 | 0 |
| 15-19 |  |  |  |  | 0 | 0 |
| 20-24 |  |  |  |  | 1 | 1 |
| 25-29 |  |  |  |  | 1 | 1 |
| 30-34 | 11 | 6 | 1 | 0 | 1 | 1 |
| 35-39 | 16 | 11 | 1 | 1 | 2 | 1 |
| 40-44 | 25 | 15 | 2 | 1 | 3 | 2 |
| 45-49 | 39 | 26 | 2 | 2 | 5 | 3 |
| 50-54 | 56 | 37 | 3 | 2 | 7 | 4 |
| 55-59 | 87 | 53 | 5 | 3 | 10 | 6 |
| 60-64 | 139 | 79 | 8 | 5 | 16 | 9 |
| 65-69 | 212 | 132 | 14 | 9 | 25 | 15 |
| 70-74 | 294 | 203 | 20 | 14 | 34 | 23 |
| 75-79 | 333 | 254 | 24 | 19 | 38 | 29 |
| 80-84 | 352 | 349 | 25 | 26 | 40 | 40 |
| 85Plus | 793 | 1,174 | 63 | 96 | 90 | 133 |

Table SI 3: Data used for Figure 4. YLL per age group and gender from exposure to PM_2.5_, NO_2_ and O_3_ for the year 2023.

| Age group | PM_2.5_ YLL | | NO_2_ YLL | | O_3_ YLL | |
| --- | --- | --- | --- | --- | --- | --- |
|  | Male | Female | Male | Female | Male | Female |
| 0-1 |  |  |  |  | 1,203 | 1,040 |
| 1-4 |  |  |  |  | 3 | 3 |
| 5-9 |  |  |  |  | 25 | 24 |
| 10-14 |  |  |  |  | 21 | 23 |
| 15-19 |  |  |  |  | 27 | 22 |
| 20-24 |  |  |  |  | 51 | 34 |
| 25-29 |  |  |  |  | 58 | 29 |
| 30-34 | 562 | 335 | 37 | 21 | 67 | 40 |
| 35-39 | 729 | 537 | 48 | 34 | 86 | 63 |
| 40-44 | 985 | 660 | 63 | 41 | 115 | 77 |
| 45-49 | 1,383 | 1,022 | 84 | 61 | 161 | 119 |
| 50-54 | 1,728 | 1,251 | 104 | 75 | 202 | 146 |
| 55-59 | 2,279 | 1,560 | 135 | 93 | 267 | 183 |
| 60-64 | 3,049 | 1,955 | 185 | 121 | 357 | 228 |
| 65-69 | 3,785 | 2,678 | 242 | 178 | 439 | 309 |
| 70-74 | 4,174 | 3,269 | 286 | 231 | 478 | 373 |
| 75-79 | 3,645 | 3,138 | 260 | 231 | 415 | 358 |
| 80-84 | 2,876 | 3,160 | 208 | 237 | 328 | 360 |
| 85Plus | 4,038 | 6,220 | 321 | 506 | 458 | 704 |

Table SI 4: Total population size, percent of population over 65 years, and between 0-14 years, exposures, PD, PD per 100,000 residents, YLL and YLL per 100,000 residents for PM_2.5_, NO_2_ and SOMO35 for countries in Europe and Israel. Data for European countries taken from Soares et al. (2022).

| Country | Population  (Thousands) | % Population  Over 65***** | % Population  between 0-14 years***** | PM_2.5_ | | | | | | NO_2_ | | | | | | SOMO35 | | | | | |
| --- | --- | --- | --- | --- | --- | --- | --- | --- | --- | --- | --- | --- | --- | --- | --- | --- | --- | --- | --- | --- | --- |
|  |  |  |  | PM_2.5_ Annual mean (ug/Sm3) | PD | PD / 100k | YLL | YLL / 100k | PD / 100k >65YO | NO_2_ Annual mean (ug/m3) | PD | PD / 100k | YLL | YLL / 100k | PD / 100k >65YO | SOMO35 (ug/m3. days) | PD | PD / 100k | YLL | YLL / 100k | PD / 100k >65YO |
| Austria | 8,901 | 19.8 | 14.4 | 9.9 | 3,200 | 36 | 30,600 | 344 | 1.82 | 14.3 | 810 | 9.1 | 7,800 | 88 | 0.46 | 4,584 | 470 | 5.3 | 4,600 | 52 | 0.27 |
| Belgium | 11,522 | 19.7 | 16.5 | 9.4 | 3,900 | 34 | 36,200 | 314 | 1.72 | 14.3 | 1,100 | 9.5 | 9,800 | 85 | 0.48 | 3,798 | 530 | 4.6 | 5,100 | 44 | 0.23 |
| Bulgaria | 6,951 | 22.4 | 14.0 | 17 | 10,600 | 152 | 107,900 | 1,552 | 6.81 | 16.7 | 1,700 | 24.5 | 17,000 | 245 | 1.09 | 2,967 | 430 | 6.2 | 4,400 | 64 | 0.28 |
| Croatia | 4,058 | 22.4 | 14.1 | 15.4 | 4,100 | 101 | 40,000 | 985 | 4.52 | 13.1 | 420 | 10.3 | 4,100 | 102 | 0.46 | 4,760 | 300 | 7.4 | 3,000 | 75 | 0.33 |
| Cyprus | 1,230 | 14.8 | 15.9 | 14 | 560 | 46 | 6,000 | 490 | 3.07 | 20.8 | 180 | 14.6 | 2,000 | 160 | 0.99 | 6,295 | 60 | 4.9 | 700 | 57 | 0.33 |
| Czechia | 10,694 | 20.6 | 16.0 | 12.5 | 6,900 | 65 | 68,700 | 643 | 3.13 | 12.5 | 740 | 6.9 | 7,300 | 68 | 0.34 | 4,252 | 620 | 5.8 | 6,300 | 59 | 0.28 |
| Denmark | 5,823 | 20.5 | 16.1 | 7.6 | 1,000 | 17 | 11,200 | 193 | 0.84 | 7.5 | 40 | 0.7 | 440 | 8 | 0.03 | 2,287 | 140 | 2.4 | 1,600 | 27 | 0.12 |
| Estonia | 1,329 | 20.6 | 16.4 | 5.4 | 60 | 5 | 690 | 52 | 0.22 | 5.8 | 10 | 0.8 | 10 | - | 0.04 | 1,468 | 30 | 2.3 | 300 | 23 | 0.11 |
| Finland | 5,525 | 23.3 | 15.2 | 4.4 | 60 | 1 | 680 | 12 | 0.05 | 6.2 | 10 | 0.2 | 80 | 2 | 0.01 | 1,365 | 80 | 1.4 | 930 | 17 | 0.06 |
| France | 65,178 | 21.7 | 17.2 | 8.6 | 16,500 | 25 | 175,800 | 270 | 1.17 | 12.2 | 4,400 | 6.8 | 47,000 | 72 | 0.31 | 4,271 | 3,100 | 4.8 | 34,100 | 52 | 0.22 |
| Germany | 83,166 | 22.4 | 14.0 | 9.1 | 28,900 | 35 | 296,300 | 356 | 1.55 | 15.2 | 10,000 | 12.0 | 102,700 | 123 | 0.54 | 4,195 | 4,600 | 5.5 | 49,100 | 59 | 0.25 |
| Greece | 10,718 | 22.8 | 13.9 | 14.5 | 8,800 | 82 | 86,200 | 804 | 3.60 | 16.9 | 1,900 | 17.7 | 18,300 | 171 | 0.78 | 6,167 | 920 | 8.6 | 9,200 | 85 | 0.38 |
| Hungary | 9,770 | 20.0 | 14.4 | 14.5 | 9,500 | 97 | 102,500 | 1,049 | 4.86 | 14.9 | 1,400 | 14.3 | 15,000 | 154 | 0.72 | 4,044 | 640 | 6.6 | 7,200 | 73 | 0.33 |
| Ireland | 4,964 | 15.1 | 19.6 | 7.1 | 490 | 10 | 5,900 | 120 | 0.65 | 7.4 | 50 | 1.0 | 580 | 12 | 0.07 | 1,908 | 70 | 1.4 | 880 | 18 | 0.09 |
| Italy | 59,641 | 24.1 | 12.4 | 15 | 52,300 | 88 | 462,300 | 775 | 3.65 | 17.7 | 11,200 | 18.8 | 98,700 | 165 | 0.78 | 6,067 | 5,100 | 8.6 | 45,900 | 77 | 0.36 |
| Latvia | 1,908 | 21.9 | 15.6 | 9.1 | 830 | 44 | 9,000 | 474 | 1.99 | 9.7 | 100 | 5.2 | 1,100 | 59 | 0.24 | 1,699 | 50 | 2.6 | 610 | 32 | 0.12 |
| Lithuania | 2,794 | 20.8 | 15.3 | 9.8 | 1,500 | 54 | 15,900 | 571 | 2.58 | 10.1 | 140 | 5.0 | 1,500 | 54 | 0.24 | 2,044 | 100 | 3.6 | 1,100 | 39 | 0.17 |
| Luxembourg | 626 | 15.0 | 15.8 | 7.3 | 80 | 13 | 810 | 129 | 0.85 | 15.8 | 50 | 8.0 | 560 | 89 | 0.53 | 4,272 | 20 | 3.2 | 250 | 40 | 0.21 |
| Malta | 515 | 19.1 | 13.1 | 10.2 | 150 | 29 | 1,700 | 338 | 1.52 | 11 | 10 | 1.9 | 140 | 26 | 0.10 | 6,592 | 30 | 5.8 | 350 | 68 | 0.30 |
| Netherlands | 17,408 | 20.3 | 15.4 | 9.1 | 5,000 | 29 | 49,800 | 286 | 1.41 | 15.9 | 1,800 | 10.3 | 18,500 | 106 | 0.51 | 3,426 | 640 | 3.7 | 6,700 | 39 | 0.18 |
| Poland | 37,958 | 18.6 | 15.2 | 16 | 36,500 | 96 | 415,700 | 1,095 | 5.18 | 13.1 | 3,400 | 9.0 | 38,500 | 101 | 0.48 | 3,216 | 1,700 | 4.5 | 20,300 | 53 | 0.24 |
| Portugal | 9,795 | 22.9 | 13.2 | 8.1 | 2,600 | 27 | 25,800 | 264 | 1.16 | 12.5 | 850 | 8.7 | 8,300 | 85 | 0.38 | 3,585 | 470 | 4.8 | 4,800 | 49 | 0.21 |
| Romania | 19,329 | 18.6 | 15.9 | 15.2 | 21,600 | 112 | 234,100 | 1,211 | 5.99 | 15.1 | 3,100 | 16.0 | 33,800 | 175 | 0.86 | 2,955 | 1,000 | 5.2 | 11,300 | 58 | 0.28 |
| Slovakia | 5,458 | 17.0 | 15.7 | 14.5 | 3,900 | 71 | 45,700 | 838 | 4.21 | 11.3 | 210 | 3.8 | 2,400 | 45 | 0.23 | 3,867 | 260 | 4.8 | 3,100 | 57 | 0.28 |
| Slovenia | 2,096 | 21.0 | 15.1 | 12.5 | 1,300 | 62 | 11,900 | 569 | 2.96 | 12.8 | 150 | 7.2 | 1,500 | 69 | 0.34 | 5,008 | 130 | 6.2 | 1,300 | 61 | 0.30 |
| Spain | 45,166 | 20.3 | 13.8 | 10 | 17,000 | 38 | 164,700 | 365 | 1.86 | 14.6 | 4,800 | 10.6 | 46,600 | 103 | 0.52 | 4,522 | 2,400 | 5.3 | 24,100 | 53 | 0.26 |
| Sweden | 10,328 | 20.2 | 17.6 | 4.8 | 370 | 4 | 3,300 | 32 | 0.18 | 6.5 | 40 | 0.4 | 380 | 4 | 0.02 | 2,181 | 240 | 2.3 | 2,200 | 22 | 0.11 |
| Albania | 2,846 | 16.7 | 16.1 | 15.6 | 3,600 | 126 | 36,900 | 1,296 | 7.59 | 12.8 | 330 | 11.6 | 3,300 | 116 | 0.70 | 5,678 | 310 | 10.9 | 3,300 | 115 | 0.65 |
| Andorra | 78 | 15.0 | 12.8 | 8.5 | 20 | 26 | 210 | 267 | 1.71 | 17.6 | 10 | 12.8 | 120 | 150 | 0.86 | 2,812 | 10 | 12.8 | 30 | 35 | 0.86 |
| Bosnia and Herzegovina | 3,825 | 18.4 | 14.9 | 25.8 | 9,200 | 241 | 91,000 | 2,379 | 13.07 | 14.1 | 610 | 15.9 | 6,100 | 158 | 0.87 | 4,047 | 300 | 7.8 | 3,100 | 81 | 0.43 |
| Iceland | 364 | 15.3 | 18.5 | 4.2 | 1 | 0 | 5 | 1 | 0.02 | 7.2 | 10 | 2.7 | 10 | 1 | 0.18 | 1,582 | 10 | 2.7 | 50 | 13 | 0.18 |
| Kosovo | 1,782 | 10.2 | 21.3 | 19.4 | 3,100 | 174 | 30,400 | 1,706 | 17.07 | 14.4 | 260 | 14.6 | 2,600 | 147 | 1.43 | 3,901 | 130 | 7.3 | 1,400 | 78 | 0.72 |
| Liechtenstein | 39 | 19.4 | 14.5 | 8.1 | 10 | 26 | 70 | 186 | 1.32 | 15.3 | 10 | 25.6 | 30 | 85 | 1.32 | 4,976 | 10 | 25.6 | 20 | 52 | 1.32 |
| Monaco | 38 | 35.9 | 13.0 | 10.5 | 20 | 53 | 160 | 407 | 1.47 | 18.1 | 10 | 26.3 | 60 | 157 | 0.73 | 6,445 | 10 | 26.3 | 30 | 78 | 0.73 |
| Montenegro | 622 | 16.6 | 15.4 | 17.4 | 920 | 148 | 9,500 | 1,531 | 8.94 | 13.7 | 90 | 14.5 | 950 | 153 | 0.87 | 4,338 | 50 | 8.0 | 560 | 90 | 0.49 |
| North Macedonia | 2,076 | 14.9 | 16.0 | 20.3 | 3,800 | 183 | 34,600 | 1,668 | 12.28 | 14.2 | 290 | 14.0 | 2,700 | 128 | 0.94 | 4,345 | 180 | 8.7 | 1,700 | 80 | 0.58 |
| Norway | 5,368 | 18.4 | 16.7 | 4.6 | 160 | 3 | 1,600 | 30 | 0.16 | 8.2 | 90 | 1.7 | 970 | 18 | 0.09 | 2,042 | 90 | 1.7 | 990 | 18 | 0.09 |
| San Marino | 35 | 20.5 | 12.7 | 12.8 | 20 | 57 | 210 | 613 | 2.79 | 13.2 | 10 | 28.6 | 20 | 69 | 1.40 | 5,387 | 10 | 28.6 | 20 | 69 | 1.40 |
| Serbia | 6,927 | 20.6 | 14.4 | 22.7 | 14,400 | 208 | 142,900 | 2,063 | 10.11 | 14.9 | 1,200 | 17.3 | 11,500 | 166 | 0.84 | 3,099 | 420 | 6.1 | 4,300 | 62 | 0.29 |
| Switzerland | 8,606 | 19.3 | 15.0 | 8.1 | 1,700 | 20 | 16,000 | 186 | 1.02 | 14.5 | 660 | 7.7 | 6,200 | 72 | 0.40 | 5,387 | 450 | 5.2 | 4,500 | 52 | 0.27 |
| Turkiye | 83,155 | 8.6 | 23.2 |  |  |  |  |  |  | 24.9 | 12,300 | 14.8 | 161,900 | 195 | 1.71 | 4,561 | 2,300 | 2.8 | 36600 | 44 | 0.32 |
| Israel** | 9,217 | 12.0 | 28.1 | 15.7 | 4,088 | 44.4 | 48,546 | 527 | 3.70 | 12.1 | 372 | 4.0 | 4,193 | 45.5 | 0.34 | 8,390 | 458 | 5.0 | 7,188 | 78 | 0.41 |

^*^ World bank data for 2022

^**^ Air quality data for Israel are for 2020


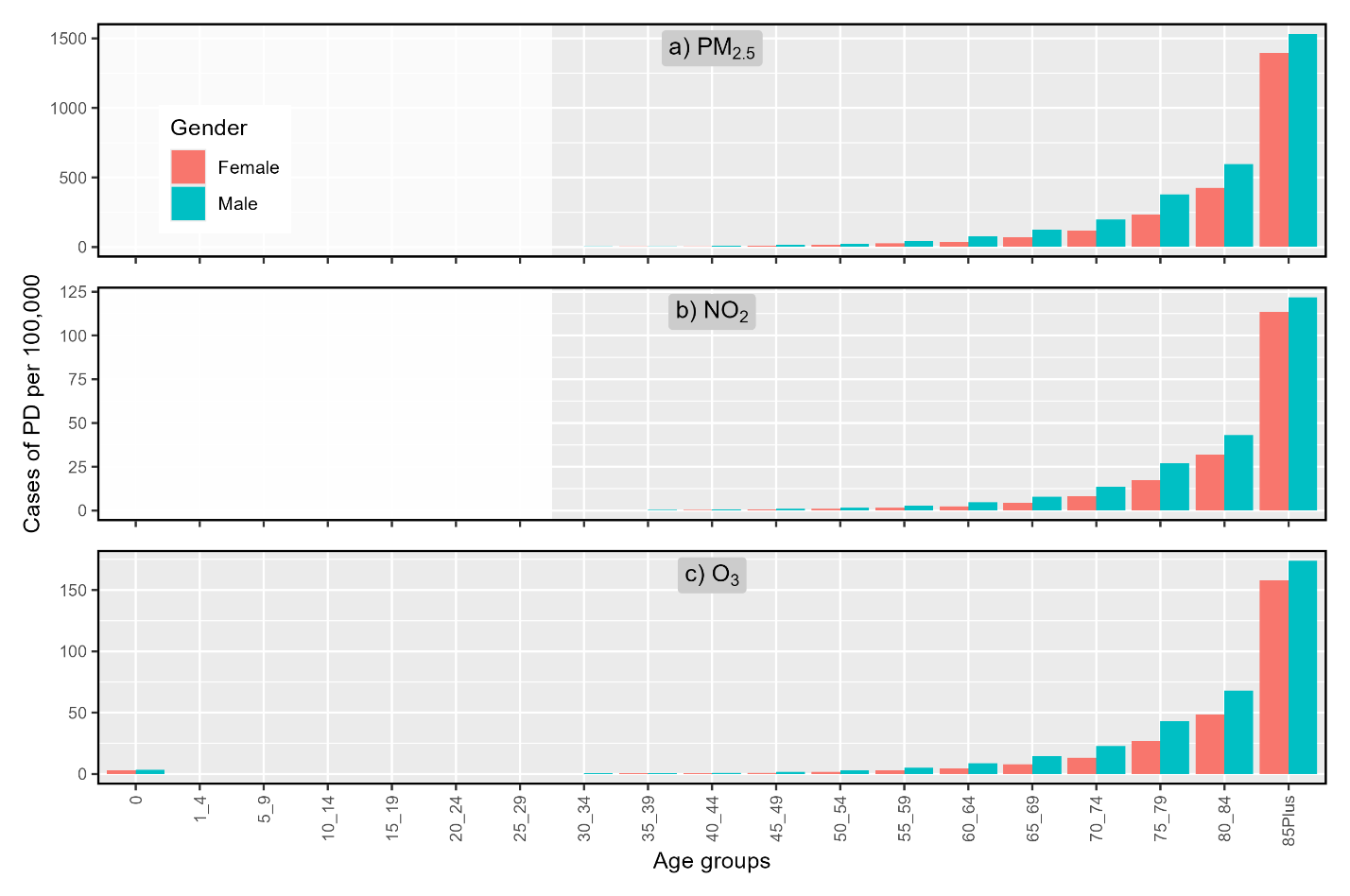


Figure SI 1: Normalized PD per 100,000 residents by age group and gender from exposure to PM_2.5_ (top), NO_2_ (center) and O_3_ (bottom) for the year 2023. Note the different scales in the y-axes.


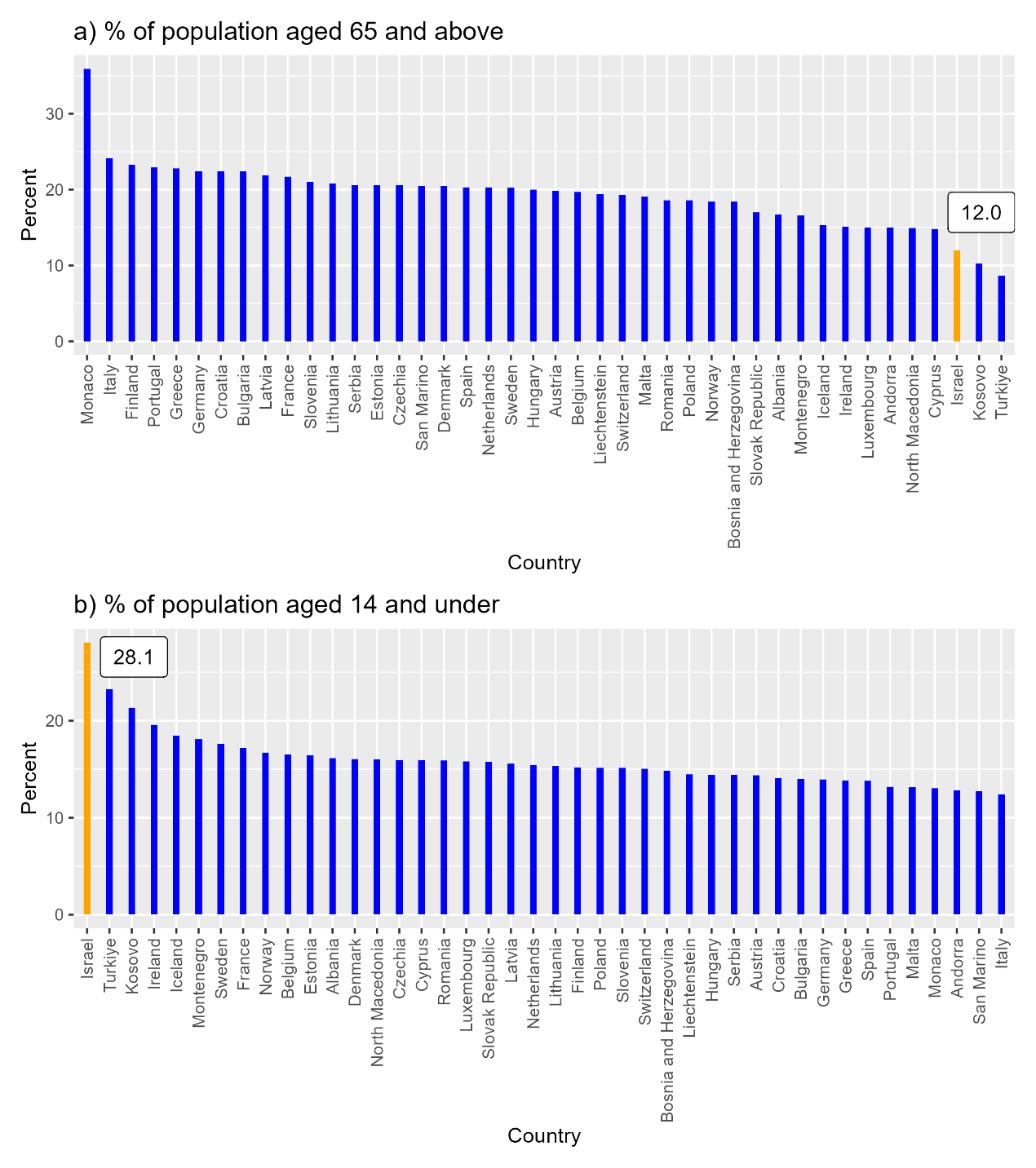


Figure SI 2: Percent of population over 65 years old (a) and population between ages 0-14 (b) for Israel (orange) and European countries. Source: World Bank data for 2022.
